# How to improve adherence of guidelines for localized testicular cancer surveillance: A Delphi consensus study

**DOI:** 10.3389/fonc.2022.1036190

**Published:** 2022-10-17

**Authors:** Angélique Da Silva, Aude Fléchon, Elodie Coquan, François Planchamp, Stéphane Culine, Thibaut Murez, Arnaud Méjean, David Pasquier, Christine Chevreau, Karim Fizazi, Antoine Thiery-Vuilemin, Florence Joly

**Affiliations:** ^1^ Centre François-Baclesse, Department of Medical Oncology, Caen, France; ^2^ Centre Léon Bérard, Department of Medical Oncology, Lyon, France; ^3^ Centre François-Baclesse, Clinical Research Unit, Caen, France; ^4^ Institut Bergonié, Clinical Research Unit, Bordeaux, France; ^5^ Hôpital Saint-Louis, Department of Medical Oncology, Paris, France; ^6^ Hôpital Lapeyronie, Department of Urology, Montpellier, France; ^7^ Georges Pompidou European Hospital, Assistance Publique Hôpitaux de Paris, Department of Urology, Paris, France; ^8^ Centre Oscar Lambret, Department of Radiation Oncology, Lille, France; ^9^ Oncopôle, Department of Medical Oncology, Toulouse, France; ^10^ Gustave Roussy, University of Paris Sud, Department of Medical Oncology, Villejuif, France; ^11^ CHRU Besancon, Hôpital Jean Minjoz, Department of Medical Oncology, Besancon, France

**Keywords:** consensus, de-escalation, delphy method, surveillance, testicular germ cell cancer

## Abstract

Stage-I testicular germ-cell tumor (TGCT) has excellent cure rates. Surveillance is fully included in patient’s management, particularly during the first years of follow-up. Surveillance guidelines differ between the academic societies, mainly concerning imaging frequency and long-term follow-up. We evaluated surveillance practice and schedules followed by French specialists and set up a DELPHI method to obtain a consensual surveillance program with an optimal schedule for patients with localized TGCT. First, an online survey on surveillance practice of stage-I TGCT based on clinical-cases was conducted among urologists, radiation-oncologists and medical-oncologists. These results were compared to ESMO/EAU and AFU guidelines. Then a panel of experts assessed surveillance proposals following a Delphi-CM. Statements were drafted after analysis of the previous survey and systematic literature review, with 2 successive rounds to reach a consensus. The study was conducted between July 2018 and May 2019. Concerning the first step: 61 participated to the survey (69% medical-oncologists, 15% urologists, 16% radiation-oncologists). About 65% of practitioners followed clinico-biological guidelines concerning 1 to 5 years of follow-up, but only 25% stopped surveillance after the 5th-year. No physician followed the EAU/ESMO guidelines of de-escalation chest imaging. Concerning the second step: 32 experts (78% medical-oncologists, 16% urologists, 6% radiation-oncologists) participated to the Delphi-CM. Thanks to Delphi-CM, a consensus was reached for 26 of the 38 statements. Experts agreed on clinico-biological surveillance modalities and end of surveillance after the 5th-year of follow-up. For seminoma, abdominal ultrasound was proposed as an option to the abdominopelvic (AP) scan for the 4th-year of follow-up. No consensus was reached regarding de-escalation of chest imaging. To conclude, the survey proved that French TGCT-specialists do not follow current guidelines. With Delphi-CM, a consensus was obtained for frequency of clinico-biological surveillance, discontinuation of surveillance after the 5th-year, stop of AP scan on the 4th-year of follow-up for seminoma. Questions remains concerning type and frequency of chest imaging.

## 1 Introduction

Testicular germ cell tumor (TGCT), including seminoma and non-seminoma, is a good prognosis cancer. It mainly affects young men with a peak of incidence around 30 years (1). Localized stage [stage IA - IB as defined in AJCC 2017 (2)] is more frequent and represent almost 70% of cases (3). After surgery, management of localized TGCT will be either surveillance alone, or adjuvant chemotherapy followed by surveillance, depending on histology, patient’s comorbidities and risk factors for recurrence.

The different French and European academic societies [European Association of Urology (EAU), French Association of Urology (AFU), European Society for Medical Oncology (ESMO)] have published recommendations concerning follow-up schemes for TGCT. Recent guidelines, suggest a de-escalation in surveillance, particularly for radiological imaging and for the surveillance rhythm after the first 2 years of follow-up (4–7). Follow-up schemes are based on expert’s opinions because of lack of clinical trial data. Guidelines differ between academic societies, as EAU and AFU recommendations for imaging surveillance (**Supplementary Table 1**). Given these differences, it induces more complexity for the practitioner to choose the follow-up scheme to apply.

Initially, we conducted this study to evaluate adherence to guidelines and to study which follow-up schemes were applied by French practitioners. Then, we applied a Delphi-consensus method (Delphi-CM) in order to obtain a consensus concerning surveillance of localized TGCT among French practitioners involved in French Urology Association (AFU) and Genito-urinary group (GETUG).

## 2 Material and methods

### 2.1 Step 1: Survey

An electronic survey was developed by the steering committee to ascertain current clinical follow-up practices. Participants were identified through the AFU and GETUG email lists consisting of physicians caring for TGCT patients. The survey was sent by email to 500 members. It was open from July to October 2018; data were collected through an online survey system.

The survey contained 23 questions divided into two sections: Part I related to demographic data of the responders and Part II asked responders to evaluate patient follow-up in four different cases. Two cases involved surveillance for seminoma with or without adjuvant chemotherapy, and two cases involved surveillance for non-seminoma in the same situations (**Supplementary Data 1**). Questionnaires were closed multiple-choice-questions, based on national (AFU) and international (EAU, ESMO) guidelines. Participants had to answer at least one question of the Part II to be included. Responses were quantitative variables. We calculated medians of each response and compared them to AFU and EAU/ESMO’s guidelines. Survey responses consistent with guidelines were considered as adherence of the recommendation. Conversely, in case of disagreement, it was considered as non-adherence. All non-adherence statements were discussed with the Delphi-CM in the step 2.

### 2.2 Step 2: Delphi consensus method

#### 2.2.1 Steering committee

The steering committee was composed of five oncologists (FJ, KF, SC, AF, AD), two urologist (AM, TM), one radiation-oncologists (DP) and a biostatistician (FP). The following steps were carried out by the steering committee including: (1) selection of expert panel; (2) generation of clinical statements based on the controversial results from the survey and on the current medical literature; (3) definition of the consensus levels and agreement according to Delphi-CM; (4) interpretation of the results; (5) final wording in the consensus document.

#### 2.2.2 Expert panel

The expert panel involved in the rating process were composed of experts in uro-oncology who were solicited within the AFU’s and the GETUG’s group. They received questionnaires by email. The nominative lists of experts who accepted to participate are provided in the acknowledgments section. Responses were collected from March to June 2019.

#### 2.2.3 Generation of statements

Based on the survey results, only follow-up practices that differed from AFU and EAU/ESMO guidelines were discussed. After literature review, 38 statements belonging to 4 major clinical situations (seminoma and non-seminoma in surveillance only or after adjuvant chemotherapy, respectively) were written by the steering committee.

#### 2.2.4 Rating and analysis of the questionnaire

For the first round: experts were asked to indicate on a scale ranging from one (totally disagree) to nine (totally agree) how the statement was relevant. For each rating lower than 7, participants were invited to justify their votes, in order to understand the reason of disagreement and to adjust the statement. A statement was defined as appropriate (approved its acceptance) if the median of all scores was ≥ 7 and there was agreement among all experts of the rating committee (range of rating 7–9 for strong consensus and 5–9 for relative consensus); or inappropriate (did not satisfied to consider) if the median of all scores was ≤ 3; or uncertain if the median of all scores was comprised between 4 and 6. After the first round, statements with strong consensus were accepted and those inappropriate were rejected. Others items were reassessed by the steering committee. For the second round: questionnaire and results of the first round were sent to all expert of the rating group. Each expert was asked to rate the questionnaire with the same scale. The same rating methodology as for the first round was applied, however appropriate statement with relative consensus were accepted.

### 2.3 Ethics approval

This study consisted of a survey of expert opinions and no patient data were collected, so no ethical approval was required to perform this study.

## 3 Results

### 3.1 Step 1: Survey

Sixty-one participants completed the survey resulting in a 12.2% response rate. Characteristics concerning the demographic data are presented in **Table 1**. Majority of them are in charge of more than 5 patients with TGCT per year. According to clinical situations, we observed a heterogeneity of follow-up during the first five years of follow-up with 30 to 50% adherence to AFU’s guidelines, 20 to 36% adherence to EAU/ESMO’s guidelines and 6 to 45% of practices not corresponding to any guidelines. **Tables 2**, **3** report the current practice of respondents compared to guidelines. After the 5th year of surveillance, only 21 to 34% of respondents declared stopping follow-up as recommended by guidelines. More than 50% of respondents affirmed to continue follow-up until the 10th year of surveillance (**Table 4**).

**Table 1:** Baseline characteristics of survey respondents and Delphi consensus method experts.

	Survey respondents			Delphi method experts	
	Total			Total	
**Baseline characteristics**	**N**	**%**		**N**	**%**
Specialty/Discipline					
Medical oncology	42	69		25	78
Urologist	9	15		5	16
Radiation-oncologists	10	16		2	6
Patient volume					
Current estimated annual new TGCT patient volume					
Less than 1	3	5			
1 to 5	17	28			
More than 5	41	67		32	100

**Table 2:** Results of survey for seminoma comparing to academic societies guidelines.

(number of times per year)						AFU^6^			EAU/ESMO^4,5,7^		Agreement between guidelines and survey
Seminoma on surveillance only	Surveillance on the 1^st^ year										
	Clinical/biological				3/3				2/2		4/2	
	Imaging		Thoracic CT scan		2				0		2	
			Abdominal CT scan or MRI		2				2		2	
	Surveillance on the 2^nd^ year										
	Clinical/biological			2/2				2/2			3/3	
	Imaging	Thoracic CT scan			2			0			2	
		Abdominal CT scan or MRI			2			2			2	
	Surveillance on the 3^rd^ year										
	Clinical/biological			2/2				2/2			1/1	
	Imaging	Thoracic CT scan			1			0			1	
		Abdominal CT scan or MRI			1			1			1	
	Surveillance on the 4^th^ year										
	Clinical/biological			1/1				1/1			1/1	
	Imaging	Thoracic CT scan			0			0			1	
		Abdominal CT scan or MRI			0			0			1	
	Surveillance on the 5^th^ year										
	Clinical/biological			1/1				1/1			1/1	
	Imaging	Thoracic CT scan			1			0			1	
		Abdominal CT scan or MRI			1			1			1	
Seminoma after adjuvant chemotherapy	Surveillance on the 1^st^ year										
	Clinical/biological			3/3				2/2			3/2	
	Imaging	Thoracic CT scan			2			0			2	
		Abdominal CT scan or MRI			2			2			2	
	Surveillance on the 2^nd^ year										
	Clinical/biological			2/2				2/2			2/2	
	Imaging	Thoracic CT scan			2			0			1	
		Abdominal CT scan or MRI			2			2			2	
	Surveillance on the 3^rd^ year										
	Clinical/biological			2/2				2/2			2/1	
	Imaging	Thoracic CT scan			1			0			1	
		Abdominal CT scan or MRI			1			1			1	
	Surveillance on the 4^th^ year										
	Clinical/biological			1/1				1/1			2/1	
	Imaging	Thoracic CT scan			0			0			1	
		Abdominal CT scan or MRI			0			0			1	
	Surveillance on the 5^th^ year										
	Clinical/biological			1/1				1/1			2/1	
	Imaging	Thoracic CT scan			1			0			1	
		Abdominal CT scan or MRI			1			1			1	

The first column contains the surveillance schedules according to the AFU’s guidelines. The second column contains the surveillance schedules according to the EAU and ESMO’s guidelines. And the third column contains results of survey, expressed as a median of each response.

**Table 3:** Results of survey for non-seminoma comparing to academic societies guidelines.

(number of times per year)				AFU^6^		EAU/ESMO^4,5,7^		Agreement between guidelines and survey
Non seminoma on surveillance only	Surveillance on the 1^st^ year							
	Clinical/biological		5/5			4/4		4/4	
	Imaging	Chest X-ray	0			2		0	
		Thoracic CT scan	2			0		2	
		Abdominal CT scan or MRI	2			2		2	
	Surveillance on the 2^nd^ year							
	Clinical/biological		4/4			4/4		4/4	
	Imaging	Chest X-ray	0			2		0	
		Thoracic CT scan	1 or 2 (if LVI +)			0		2	
		Abdominal CT scan or MRI	1 or 2 (if LVI +)			1		2	
	Surveillance on the 3^rd^ year							
	Clinical/biological		2/2			2/2		2/2	
	Imaging	Chest X-ray	0			0 or 1		0	
		Thoracic CT scan	1			0		1	
		Abdominal CT scan or MRI	1			1		1	
	Surveillance on the 4^th^ year							
	Clinical/biological		1 or 2/1 or 2			1 or 2/1 or 2		2/2	
	Imaging	Chest X-ray	0			0		0	
		Thoracic CT scan	0			0		1	
		Abdominal CT scan or MRI	0			0		1	
	Surveillance on the 5^th^ year							
	Clinical/biological		1 or 2/1 or 2			1 or 2/1 or 2		2/2	
	Imaging	Chest X-ray	0			1 if LVI +		0	
		Thoracic CT scan	1			0		1	
		Abdominal CT scan or MRI	1			1		1	
Non seminoma after adjuvant chemotherapy	Surveillance on the 1^st^ year							
	Clinical/biological		5/5			4/4		4/4	
	Imaging	Chest X-ray	0			1 or 2		0	
		Thoracic CT scan	2			0		2	
		Abdominal CT scan or MRI	2			1 or 2		2	
	Surveillance on the 2^nd^ year							
	Clinical/biological		4/4			4/4		2/4	
	Imaging	Chest X-ray	0			1		0	
		Thoracic CT scan	1 or 2 (if LVI +)			0		2	
		Abdominal CT scan or MRI	1 or 2 (if LVI +)			1		2	
	Surveillance on the 3^rd^ year							
	Clinical/biological		2/2			2/2		2/2	
	Imaging	Chest X-ray	0			1		0	
		Thoracic CT scan	1			0		1	
		Abdominal CT scan or MRI	1			1		1	
	Surveillance on the 4^th^ year							
	Clinical/biological		1 or 2/1 or 2			2/2		2/2	
	Imaging	Chest X-ray	0			1		0	
		Thoracic CT scan	0			0		1	
		Abdominal CT scan or MRI	0			0		1	
	Surveillance on the 5^th^ year							
	Clinical/biological		1 or 2/1 or 2			2/2		2/2	
	Imaging	Chest X-ray	0			1		0	
		Thoracic CT scan	1			0		1	
		Abdominal CT scan or MRI	1			1		1	

LVI, lymphovascular invasion. The first column contains the surveillance schedules according to the AFU’s guidelines. The second column contains the surveillance schedules according to the EAU and ESMO’s guidelines. And the third column contains results of survey, expressed as a median of each response.

**Table 4:** Current practice of respondents after the 5^th^ year of follow-up.

Clinical situation	Response	Total N (%)
Seminoma		
surveillance alone		
	Stop surveillance	21 (34)
	Continuation of surveillance for 10 years	34 (56)
	Continuation of surveillance life-long	4 (7)
	Abstention	2 (3)
surveillance after chemotherapy		
	Stop surveillance	16 (26)
	Continuation of surveillance for 10 years	37 (61)
	Continuation of surveillance life-long	5 (8)
	Abstention	3 (5)
Non seminoma		
surveillance alone		
	Stop surveillance	14 (23)
	Continuation of surveillance for 10 years	39 (64)
	Continuation of surveillance life-long	4 (6.5)
	Abstention	4 (6.5)
surveillance after chemotherapy		
	Stop surveillance	13 (22)
	Continuation of surveillance for 10 years	38 (62)
	Continuation of surveillance life-long	5 (8)
	Abstention	5 (8)

### 3.2 Step 2: Delphi consensus method

#### 3.2.1 Expert panel

Thirty-two experts participated to the Delphi-CM. Characteristics of the expert panel are reported on **Table 1**.

#### 3.2.2 First round

**Figure 1:**
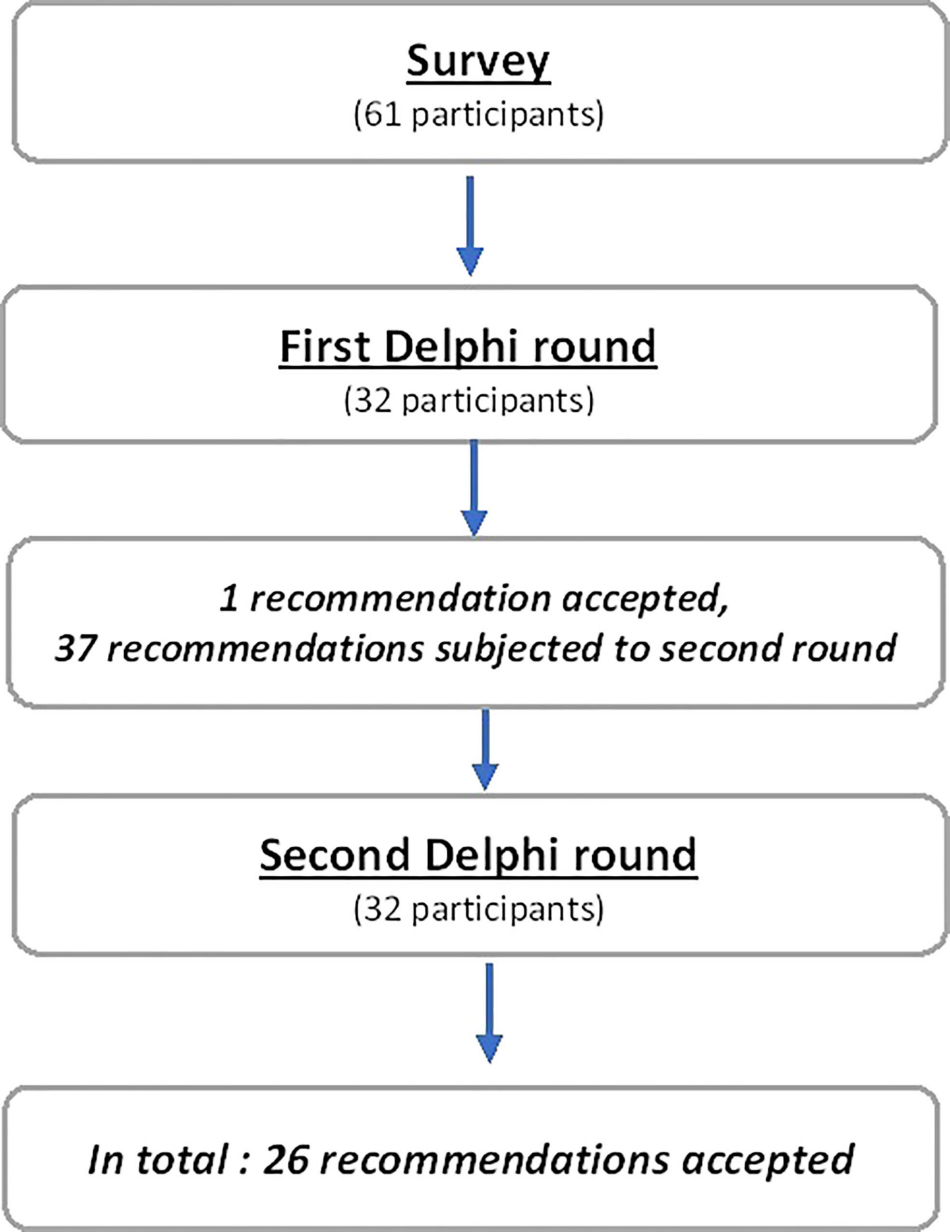
Flow chart.

The first questionnaire consisted of 38 ranking items. After round 1, a strong consensus was obtained on 1 item (2.6%), relative consensus for 1 item (2.6%), 36 items were judged uncertain (94.8%) and none were judged inappropriate (**Supplementary Table 2**). Hence, a total of 37 items were proposed for new rating (**Figure 1**).

#### 3.2.3 Second round

After the second round of rating, 4 items were judged appropriate and 33 items were uncertain. According to the Delphi-CM, we cancelled two extreme values of vote for each uncertain item. Hence, 20 additional items were accepted: 1 with strong consensus and 19 with relative consensus (**Table 5**).

**Table 5:** Results of the rating process following the second round of Delphi consensus method.

		Criterion	N	Med.	N° of responses									C
					1	2	3	4	5	6	7	8	9	
**Seminoma**	surveillance alone	1.On the 1st year: Physical examination and biological markers 3 times per year	32	9		1				1	3	8	19	R
		2.On the 2nd year: Physical examination and biological markers 2 times per year												S
		3.On the 3rd year: Physical examination 2 times per year, with one optional	32	9	1	1	5		2		2	4	17	N
		4.On the 3rd year: Biological markers 2 times per year	32	9		2	2	1			4	5	18	N
		5.No systematic chest imaging	32	7	1	1	5	3	3	2	4	5	8	N
		6.On the 4^th^ year, no systematic imaging, ultrasound abdominopelvic in option	32	8		1		1	2	1	6	9	12	R
		7.Stop surveillance after 5 years of follow-up	32	9			1		2	2	3	5	19	R
	surveillance after chemotherapy	8.On the 1st year: Physical examination and biological markers 2-3 times per year	32	9			2				5	7	18	S
		9.No systematic chest imaging	32	8		1	1	4	3		3	8	12	N
		10.On the 3rd year: Physical examination 1-2 times per year	32	9				1	1		7	6	17	R
		11.On the 4th and 5th year: Biological markers 1 time per year	32	9						1	3	7	21	R
		12.On the 4th year, no systematic imaging, ultrasound abdominopelvic in option	32	8			1		2		8	11	10	R
		13.Stop surveillance after 5 years of follow-up	32	9				1	1	2	3	8	17	R
**Non-seminoma**	surveillance alone	14.On the 1st year: Physical examination and biological markers 4 times per year (relevance)	32	9	1	1			1		4	6	19	R
		15.On the 1st year: Physical examination and biological markers 4 times per year (feasibility)	32	9	1		1			1	6	6	17	R
		16.On the 2nd year: Physical examination and biological markers 4 times per year (relevance)	32	9		1	1		4		3	6	17	R
		17.On the 2nd year: Physical examination and biological markers 4 times per year (feasibility)	32	8	1			1	2	1	6	6	15	R
		18.On the 4th and 5th year: Physical examination and biological markers 1 time per year	32	8			1			1	5	10	15	R
		19.On the 1st and 2nd year: Thoracic scanner for chest imaging	32	8.5	1					2	4	9	16	R
		20.On the 1st and 2nd year: chest imaging 2 times per year	32	8.5	2					2	4	8	16	R
		21.If LVI negative, no systematic chest imaging after the 3rd year	32	8			2	1	2	1	5	9	12	N
		22.On the 2nd year, if LVI negative: abdominopelvic imaging 1 time per year	32	8	1		2		1	1	5	9	13	N
		23.On the 4th year: no systematic imaging	32	8		1		2	3	2	2	11	11	N
		24.Stop surveillance after 5 years of follow-up	32	9			1		2	1	2	8	18	R
	Non-seminoma on surveillance after chemotherapy	25.On the 1st year: Physical examination and biological markers 4 times per year (relevance)	32	8	3	2			1		3	9	14	N
		26.On the 1st year: Physical examination and biological markers 4 times per year (feasibility)	32	8	1			1	1		6	9	14	R
		27.On the 2nd year: Physical examination and biological markers 4 times per year (relevance)	32	8	2	1	1		2	2	2	10	12	N
		28.On the 2nd year: Physical examination and biological markers 4 times per year (feasibility)	32	8	2		1	1		1	5	10	12	N
		29.On the 4th and 5th year, if LVI negative, physical examination and biological markers 1 time per year	32	9						2	1	12	17	R
		30.On the 4th and 5th year, if LVI positive, physical examination and biological markers 2 times per year	32	8	1					2	4	11	14	R
		31.Chest x-ray instead of thoracic scanner	32	6.5	5	3	1	3	2	2	3	6	7	N
		32.If LVI negative, chest imaging 1-2 times per year on the 1^st^ year then 1 time per year on the 2^nd^ year	32	9	1		1	1		1	1	10	17	N
		33.On the 1st and 2nd year, if LVI positive, chest imaging 2 times per year	32	8.5	1				1	1	2	11	16	R
		34.After the 3rd year, chest imaging 1 time per year	32	9	1					1	2	10	18	R
		35.If LVI negative, abdominopelvic imaging 1-2 times per year on the 1^st^ year then 1 time per year on the 2^nd^ year	32	8.5					1	1	4	10	16	R
		36.On the 1st and 2nd year, if LVI positive, abdominopelvic imaging 2 times per year	32	9						1	1	13	17	R
		37.On the 4th year: no systematic imaging	32	8	1		1		2	2	2	11	13	R
		38.Stop surveillance after 5 years of follow-up	32	9	1		1		1	1	2	7	19	R

Experts were asked to rate each item according to its relevance for prescription (C, consensus; S, strong; R, Relative; N, absence of consensus).

#### 3.2.4 Item with consensus

In total, strong consensus was obtained for 2 items (5.2%) concerning clinical and biological examination for seminoma. Relative consensus was reached for 24 items (63.2%) (**Table 5**). Eleven items concerned clinical and biological examination for seminoma (n=3) and non-seminoma (n=8) and 4 relating to discontinuation of surveillance after the 5th-year. Proposals on decrease of imaging have been accepted for seminoma [stop of abdomino-pelvic (AP) scan on 4th-year for seminoma (n=2)] and non-seminoma [no systematic imaging on the 4th-year after adjuvant chemotherapy (n=1)]. Concerning chest imaging, for non-seminoma, experts agreed to realize exams two times per year on the first two years (n=2), with CT-scan rather than chest X-ray in case of surveillance only (n=1) and to continue chest-imaging one time per year from the 3rd-year (n=1). Concerning AP-scan, for non-seminoma, it could be done 1-2 times (1st-year) and one time (2nd-year), in the absence of lymphovascular invasion (LVI) (n=1). If LVI, AP-scan would be done 2 times a year on the first two years (n=1).

#### 3.2.5 Item lacking consensus

Twelve items (31.6%) lacked consensus (**Table 5**). Five items concerning frequency of clinical and biological exams. Physicians disagreed with discontinuing chest imaging surveillance in seminomas (n=2). They did not accept to decrease chest imaging surveillance in non-seminoma (n=3), to reduce AP-scan on the 2nd-year of follow-up for LVI negative non-seminoma (n=1) and to stop doing scanner on the 4th-year of follow-up for non-seminoma on surveillance alone (n=1).

## 4 Discussion

Our study highlights the low-adherence of French practitioners to academic societies’ guidelines explaining a heterogeneity of practices. These differences mainly concerned frequencies of physical/biological examination and imaging, particularly methods for chest surveillance. In our study we described less than 50% of adherence to guidelines and up to 45% of practices did not corresponding to any of the guidelines. In a second time, from these conflicting situations between practices and recommendations, we performed a Delphi-CM to obtain a consensus of follow-up for TGCT. After two rounds, we reached consensus for 68.4% of statements.

Our results stress that physician adherence is critical to successful application of the recommendations. Non–guideline adherence for TGCT patients is common, most frequently in the form of inappropriate imaging (8). There are many barriers to guideline adherence including lack of awareness, lack of familiarity and lack of agreement (9, 10).

Non–guideline adherence for TGCT patients was associated with inferior global quality-of-life (11). As TGCT survival improves, quality of care such as reducing treatment and monitoring exams toxicity is one of the main objectives of the future years (12).

Due to the lack of strong scientific data, some items issued from the international guidelines have been elaborated issued from experts’ opinions, particularly for the imaging surveillance. In our survey, on the whole, the clinico-biological surveillance was in accordance to guidelines. The main disagreements concerned imaging exams. In retrospective study of TGCT patients on surveillance alone, adherence to imaging was shown to be inconsistent with guidelines (13). Thanks to the Delphi-CM, experts agreed on several proposals on imaging, as stop of imaging on the 4th-year. Experts did not agree on the decrease in chest imaging as suggest by last guidelines, whether for stopping the chest CT-scan in the follow-up of seminoma or performing a chest X-ray in non-seminoma. This lack of consensus explains the lack of adhesion on the guidelines as experts do not seem to be agree with them. This discrepancy can be explained by the limited evidence regarding pulmonary recurrence. In retrospective studies, it was showed that all relapses were detected by abdominopelvic imaging or markers elevation (14, 15). Thoracic recurrence was always associated with another abnormality (15). Moreover, survival of TGCT patients is good with a 5-year relapse-free survival of around 90% (16). These results were recently confirmed by the analysis of the SWENOTECA cohort. Tandstad et al. reported for clinical stage I TGCT 7.9% of relapse of which 1.9% occurred within the first 5 years. Only 1.5% of relapse occurred beyond the first 5 years (17).

Although there is a scientific rationale for decrease in chest imaging, it seems that practitioners are not ready to implement it in their practices. Performing repeated imaging in these young patients can have clinical impact such as an increase of the risk of radiation secondary cancers and kidney failure induced by contrast products (18–21). One of the options suggested by experts in our study, to limit radiation, was to perform a low-dose thoracic scanner for thoracic surveillance. Although the dose delivered during a low-dose scanner is lower than conventional scanner, it is still 70 to 250 times higher than dose received during a chest X-ray (22), but it could be an acceptable option.

Regarding the cessation of follow-up at 5 years, the majority of survey participants reported a continuation to 10 years. Majority of them reported that they pursued surveillance beyond 5 years because all recommendations were not clear on it. After implementation of the Delphi-CM, the experts agreed on a cessation at 5 years, in accordance with literature and EAU/ESMO guidelines (5, 23, 24).

To our knowledge this is the first report of the use of the Delphi-CM for the application in routine of different national and international recommendations for TGCT follow-up adapted to the opinion of TGCT practitioners.

Nevertheless, this study has some limitations. As purposive sampling was used (and participants were, therefore, not randomly selected), representativeness cannot be assured. However, using AFU’s and GETUG’s mailing list, we targeted different French practitioners who follow TGCT patients. Moreover, the whole premise behind the Delphi theory is that the panel members are in fact experts in their field, therefore, yielding results of increased accuracy, instead of selecting a representative sample of the population. Only 12.2% of participants responded to the survey. This could have affected the potential for guidelines’ adherence as well as the amount of data analyzed. However this participation rate corresponds to that usually found using an online questionnaire (25). With 32 experts, the size of our panels for the Delphi-CM is in accordance with the published recommendations relative to the required size of a panel used for being representative (26, 27).

## 5 Conclusion

French and European guidelines concerning the follow-up of the TGCT patients are different. Thanks to the Delphi-CM, an expert consensus was obtained for frequency of clinico-biological surveillance, stop of AP scan on the 4th year of follow-up for seminoma and discontinuation of surveillance after the 5th year. The continuation of this project is to spread these results in order to inform as many practitioners as possible to increase the adherence to the guidelines.

## Data availability statement

The raw data supporting the conclusions of this article will be made available by the authors, without undue reservation.

## Author contributions

FJ, AF and ADS conceived of the presented idea. ADS and FP verified the analytical methods. ADS performed the calculations. EC and FJ helped supervise the project. ADS, EC and FJ wrote the paper with input from all authors. All authors contributed to the article and approved the submitted version.

## Funding

The study was supported by Centre François Baclesse (Caen Normandie, France).

## Acknowledgments

The authors are grateful to the panelists for their participation to the study: Damien Pouessel, Constance Thibault, Jérôme Rigaud, François Audenet, Jean-Pierre Lotz, Pierre Clavere, Antoine Angelergues, Nadine Houede, Catherine Becht, Carolina Saldana, Cédric Lebacle, Olivier Huillard, Diego Tosi, Camille Serrate, Yohann Loriot, Géraldine Pignot, Johann Barkatz, Giulia Baciarello, Ali Hasbini, Christine Chevreau, Emmanuelle Bompas, Mouna Ayadi, Sylvestre Le Moullec, Antoine Thiery-Vuillemin, Jonathan Olivier, Franck Priou, Philippe Barthelemy, Sylvain Ladoire, Elouen Boughalem, Lionnel Geoffrois, Mathieu Laramas, Eric Voog, Loic Mourey, Friederike Schlürmann.

## Conflict of interest

The authors declare that the research was conducted in the absence of any commercial or financial relationships that could be construed as a potential conflict of interest.

## Publisher’s note

All claims expressed in this article are solely those of the authors and do not necessarily represent those of their affiliated organizations, or those of the publisher, the editors and the reviewers. Any product that may be evaluated in this article, or claim that may be made by its manufacturer, is not guaranteed or endorsed by the publisher.

## Abbreviations

AFU, French Association of Urology; EAU, European Association of Urology; ESMO, European Society for Medical Oncology; TGCT, testicular germ cell tumor.
