# Supplementary material for: How to improve adherence of guidelines for localized testicular cancer surveillance: A Delphi consensus study
Source: Front Oncol. 2022 Oct 17;12:1036190. doi: 10.3389/fonc.2022.1036190 (PMC9619048; doi:10.3389/fonc.2022.1036190)
Supplement: Supplementary file 1 [file DataSheet_1.docx]

**Clinical case N° 1/4 :**

Mr X 30 years old, with no medical history, consulted for a left testicular mass. An enlarged left orchiectomy was performed. Histological results were in favor of 2cm seminomatous germ cell tumor, limited to the testis, without invasion of the rete testis. No metastasis were found on CT scan and the postoperative tumour markers (hCG, a-foetoprotein (AFP) and lactate dehydrogenase) were normal, either a pure seminoma pT1N0M0S0. The medical staff proposed surveillance.

What are the surveillance modalities?

1 – On the first year, which clinical, biological and imaging surveillance would you suggest for this patient?

|  | Not done | 2 times per year | 3 times per year | 4 times per year |
| --- | --- | --- | --- | --- |
| Tumour markers |  |  |  |  |
| Physical examination |  |  |  |  |
| Thoracic CT scan |  |  |  |  |
| Abdominal CT scan or MRI |  |  |  |  |

other proposal:

2 – On the second year, which clinical, biological and imaging surveillance would you suggest for this patient?

|  | Not done | 1 time per year | 2 times per year | 3 times per year |
| --- | --- | --- | --- | --- |
| Tumour markers |  |  |  |  |
| Physical examination |  |  |  |  |
| Thoracic CT scan |  |  |  |  |
| Abdominal CT scan or MRI |  |  |  |  |

other proposal:

3 - From the third to the fifth year, which clinical, biological and imaging surveillance would you suggest for this patient?

|  | Not done | 1 time per year | 2 times per year |
| --- | --- | --- | --- |
| Tumour markers |  |  |  |
| Physical examination |  |  |  |
| Thoracic CT scan |  |  |  |
| Abdominal CT scan or MRI |  |  |  |

other proposal:

4 – Since the sixth year, which surveillance would you suggest for this patient?

| Stop the surveillance |  |
| --- | --- |
| Continued surveillance for up to 10 years |  |
| Continued lifetime surveillance |  |
| other proposal : |  |

**Clinical case N° 2/4 :**

Mr Y 27 years old, with no medical history, consulted for a left testicular mass. An enlarged left orchiectomy was performed. Histological results were in favor of 6cm seminomatous germ cell tumor, with invasion of the spermatic cord and lympho-vascular invasion. No metastasis were found on CT scan and the postoperative tumour markers (hCG, a-foetoprotein (AFP) and lactate dehydrogenase) were normal, either a pure seminoma pT3N0M0S0. The medical staff proposed adjuvant chemotherapy with CARBOPLATINE AUC7, follow by surveillance.

What are the surveillance modalities ?

1 – On the first year, which clinical, biological and imaging surveillance would you suggest for this patient?

|  | Not done | 1 time per year | 2 times per year | 3 times per year |
| --- | --- | --- | --- | --- |
| Tumour markers |  |  |  |  |
| Physical examination |  |  |  |  |
| Thoracic CT scan |  |  |  |  |
| Abdominal CT scan or MRI |  |  |  |  |

other proposal:

2 – On the second year, which clinical, biological and imaging surveillance would you suggest for this patient?

|  | Not done | 1 time per year | 2 times per year |
| --- | --- | --- | --- |
| Tumour markers |  |  |  |
| Physical examination |  |  |  |
| Thoracic CT scan |  |  |  |
| Abdominal CT scan or MRI |  |  |  |

other proposal:

3 - From the third to the fifth year, which clinical, biological and imaging surveillance would you suggest for this patient?

|  | Not done | 1 time per year | 2 times per year |
| --- | --- | --- | --- |
| Tumour markers |  |  |  |
| Physical examination |  |  |  |
| Thoracic CT scan |  |  |  |
| Abdominal CT scan or MRI |  |  |  |

other proposal:

4 – Since the sixth year, which surveillance would you suggest for this patient?

| Stop the surveillance |  |
| --- | --- |
| Continued surveillance for up to 10 years |  |
| Continued lifetime surveillance |  |
| other proposal : |  |

**Clinical case N° 3/4 :**

Mr B 29 years old, with no medical history, consulted for a left testicular mass. An enlarged left orchiectomy was performed. Histological results were in favor of 2cm non seminomatous germ cell tumor with yolk tumor and choriocarcinoma limited to the testis, without lympho-vascular invasion. No metastasis were found on CT scan and the postoperative tumour markers (hCG, a-foetoprotein (AFP) and lactate dehydrogenase) were normal, either a non-seminoma pT1N0M0S0. The medical staff proposed surveillance.

What are the surveillance modalities?

1 – On the first year, which clinical, biological and imaging surveillance would you suggest for this patient?

|  | Not done | 2 times per year | 3 times per year | 4 times per year | 5 times per year | 6 times per year |
| --- | --- | --- | --- | --- | --- | --- |
| Tumour markers |  |  |  |  |  |  |
| Physical examination |  |  |  |  |  |  |
| Chest X-ray |  |  |  |  |  |  |
| Thoracic CT scan |  |  |  |  |  |  |
| Abdominal CT scan or MRI |  |  |  |  |  |  |

other proposal:

2 – On the second year, which clinical, biological and imaging surveillance would you suggest for this patient?

|  | Not done | 1 time per year | 2 times per year | 4 times per year |
| --- | --- | --- | --- | --- |
| Tumour markers |  |  |  |  |
| Physical examination |  |  |  |  |
| Chest X-ray |  |  |  |  |
| Thoracic CT scan |  |  |  |  |
| Abdominal CT scan or MRI |  |  |  |  |

other proposal:

3 - From the third to the fifth year, which clinical, biological and imaging surveillance would you suggest for this patient?

|  | Not done | 1 time per year | 2 times per year | 3 times per year |
| --- | --- | --- | --- | --- |
| Tumour markers |  |  |  |  |
| Physical examination |  |  |  |  |
| Chest X-ray |  |  |  |  |
| Thoracic CT scan |  |  |  |  |
| Abdominal CT scan or MRI |  |  |  |  |

other proposal:

4 – Since the sixth year, which surveillance would you suggest for this patient?

| Stop the surveillance |  |
| --- | --- |
| Continued surveillance for up to 10 years |  |
| Continued lifetime surveillance |  |
| other proposal : |  |

**Clinical case N° 4/4 :**

Mr Z 32 years old, with no medical history, consulted for a left testicular mass. An enlarged left orchiectomy was performed. Histological results were in favor of 5cm mixed non seminomatous germ cell tumor : embryonal carcinoma on 70% and teratoma on 30%, with invasion of the scrotum and lympho-vascular invasion No metastasis were found on scanner and the postoperative tumour markers (hCG, a-foetoprotein (AFP) and lactate dehydrogenase) were normal, either a non-seminoma pT4N0M0S0. The medical staff proposed adjuvant chemotherapy with Bleomycin, Etoposide and Cisplatin (BEP), follow by surveillance.

What are the surveillance modalities?

1 – On the first year, which clinical, biological and imaging surveillance would you suggest for this patient?

|  | Not done | 1 time per year | 2 times per year | 4 times per year | 5 times per year | 6 times per year |
| --- | --- | --- | --- | --- | --- | --- |
| Tumour markers |  |  |  |  |  |  |
| Physical examination |  |  |  |  |  |  |
| Chest X-ray |  |  |  |  |  |  |
| Thoracic CT scan |  |  |  |  |  |  |
| Abdominal CT scan or MRI |  |  |  |  |  |  |

other proposal:

2 – On the second year, which clinical, biological and imaging surveillance would you suggest for this patient?

|  | Not done | 1 time per year | 2 times per year | 4 times per year |
| --- | --- | --- | --- | --- |
| Tumour markers |  |  |  |  |
| Physical examination |  |  |  |  |
| Chest X-ray |  |  |  |  |
| Thoracic CT scan |  |  |  |  |
| Abdominal CT scan or MRI |  |  |  |  |

other proposal:

3 - From the third to the fifth year, which clinical, biological and imaging surveillance would you suggest for this patient?

|  | Not done | 1 time per year | 2 times per year |
| --- | --- | --- | --- |
| Tumour markers |  |  |  |
| Physical examination |  |  |  |
| Chest X-ray |  |  |  |
| Thoracic CT scan |  |  |  |
| Abdominal CT scan or MRI |  |  |  |

other proposal:

4 – Since the sixth year, which surveillance would you suggest for this patient?

| Stop the surveillance |  |
| --- | --- |
| Continued surveillance for up to 10 years |  |
| Continued lifetime surveillance |  |
| other proposal : |  |
