# Supplementary material for: How to improve adherence of guidelines for localized testicular cancer surveillance: A Delphi consensus study
Source: Front Oncol. 2022 Oct 17;12:1036190. doi: 10.3389/fonc.2022.1036190 (PMC9619048; doi:10.3389/fonc.2022.1036190)
Supplement: Supplementary file 2 [file Table_1.docx]

**Supplementary Table 1 : Surveillance guidelines recommended by different European academic societies.**

| **(number of times per year)** | | | | **AFU** | **EAU / ESMO** | **Agreement between guidelines** |
| --- | --- | --- | --- | --- | --- | --- |
| **Seminoma** | **Surveillance on the 1^st^ year** | | |  | |  |
|  |  | Clinical and biological |  | 3 | 2 |  |
|  |  | Imaging | Thoracic CT scan | 2 | 0 |  |
|  |  |  | Abdominal CT scan or MRI | 2 | 2 | X |
|  | **Surveillance on the 2^nd^ year** | | |  | |  |
|  |  | Clinical and biological |  | 2 | 2 | X |
|  |  | Imaging | Thoracic CT scan | 2 | 0 |  |
|  |  |  | Abdominal CT scan or MRI | 2 | 2 | X |
|  | **Surveillance on the 3^rd^ year** | | |  | |  |
|  |  | Clinical and biological |  | 2 | 2 | X |
|  |  | Imaging | Thoracic CT scan | 1 | 0 |  |
|  |  |  | Abdominal CT scan or MRI | 1 | 1 | X |
|  | **Surveillance on the 4^th^ year** | | |  | |  |
|  |  | Clinical and biological |  | 1 | 1 | X |
|  |  | Imaging | Thoracic CT scan | 0 | 0 | X |
|  |  |  | Abdominal CT scan or MRI | 0 | 0 | X |
|  | **Surveillance on the 5^th^ year** | | |  | |  |
|  |  | Clinical and biological |  | 1 | 1 | X |
|  |  | Imaging | Thoracic CT scan | 1 | 0 |  |
|  |  |  | Abdominal CT scan or MRI | 1 | 1 | X |
|  | **Surveillance after the 5^th^ year** | | | Stop | Stop | X |
|  |  | | |  |  |  |
| **Non seminoma** | **Surveillance on the 1^st^ year** | | |  | |  |
|  |  | Clinical and biological |  | 5 | 4 |  |
|  |  | Imaging | Chest X-ray | 0 | 1 or 2 |  |
|  |  |  | Thoracic CT scan | 2 | 0 |  |
|  |  |  | Abdominal CT scan or MRI | 2 | 1 or 2 |  |
|  | **Surveillance on the 2^nd^ year** | | |  | |  |
|  |  | Clinical and biological |  | 4 | 4 | X |
|  |  | Imaging | Chest X-ray | 0 | 1 or 2 |  |
|  |  |  | Thoracic CT scan | 1 or 2 (if LVI +) | 0 |  |
|  |  |  | Abdominal CT scan or MRI | 1 or 2 (if LVI +) | 1 |  |
|  | **Surveillance on the 3^rd^ year** | | |  | |  |
|  |  | Clinical and biological |  | 2 | 2 | X |
|  |  | Imaging | Chest X-ray | 0 | 0 or 1 |  |
|  |  |  | Thoracic CT scan | 1 | 0 |  |
|  |  |  | Abdominal CT scan or MRI | 1 | 0 or 1 |  |
|  | **Surveillance on the 4^th^ year** | | |  | |  |
|  |  | Clinical and biological |  | 1 or 2 (if LVI +) | 1 or 2 |  |
|  |  | Imaging | Chest X-ray | 0 | 0 or 1 |  |
|  |  |  | Thoracic CT scan | 0 | 0 | X |
|  |  |  | Abdominal CT scan or MRI | 0 | 0 | X |
|  | **Surveillance on the 5^th^ year** | | |  | |  |
|  |  | Clinical and biological |  | 1 or 2 (if LVI +) | 1 or 2 |  |
|  |  | Imaging | Chest X-ray | 0 | 0 or 1 |  |
|  |  |  | Thoracic CT scan | 1 | 0 |  |
|  |  |  | Abdominal CT scan or MRI | 1 | 1 |  |
|  | **Surveillance after the 5^th^ year** | | | Stop | Stop | X |
|  |  | | |  |  |  |
| CT = computed tomography; LVI = lymphovascular invasion; + = positive | | | | | |  |
