# Supplementary material for: How to improve adherence of guidelines for localized testicular cancer surveillance: A Delphi consensus study
Source: Front Oncol. 2022 Oct 17;12:1036190. doi: 10.3389/fonc.2022.1036190 (PMC9619048; doi:10.3389/fonc.2022.1036190)
Supplement: Supplementary file 3 [file Table_2.docx]

**Supplementary table 2: Results of the rating process following the first round**

|  |  | Criterion | N | Med. | N° of responses | | | | | | | | | C |
| --- | --- | --- | --- | --- | --- | --- | --- | --- | --- | --- | --- | --- | --- | --- |
|  |  |  |  |  | 1 | 2 | 3 | 4 | 5 | 6 | 7 | 8 | 9 |  |
| Seminoma | surveillance alone | 1. On the 1^st^ year : Physical examination and biological markers 3 times per year | 32 | 8 | 1 |  | 1 |  |  |  | 7 | 10 | 13 | N |
|  |  | 1. On the 2^nd^ year : Physical examination and biological markers 2 times per year | 32 | 9 |  |  |  |  |  |  | 5 | 9 | 18 | S |
|  |  | 1. On the 3^rd^ year : Physical examination 1 time per year | 32 | 8 | 1 | 1 | 1 |  | 6 | 1 | 3 | 9 | 10 | N |
|  |  | 1. On the 3^rd^ year : Biological markers 2 times per year | 32 | 8 | 2 | 1 | 2 | 1 | 2 |  | 5 | 8 | 11 | N |
|  |  | 1. No systematic chest imaging | 32 | 5 | 1 | 3 | 3 | 3 | 7 |  | 5 | 5 | 5 | N |
|  |  | 1. On the 4^th^ year, ultrasound abdominopelvic instead of abdominopelvic scanner | 32 | 7 | 1 |  | 3 | 1 | 6 | 2 | 5 | 3 | 11 | N |
|  |  | 1. Stop surveillance after 5 years of follow-up | 32 | 8 |  | 1 |  | 2 | 3 | 1 | 5 | 7 | 13 | N |
|  | surveillance after chemotherapy | 1. On the 1st year : Physical examination and biological markers 3 times per year | 32 | 9 | 1 | 1 | 1 |  |  |  | 6 | 5 | 18 | N |
|  |  | 1. No systematic chest imaging | 32 | 7 | 1 | 2 | 3 | 1 | 5 |  | 7 | 4 | 9 | N |
|  |  | 1. On the 3rd year : Physical examination 1 time per year | 32 | 9 |  |  | 1 | 2 | 2 |  | 3 | 7 | 17 | N |
|  |  | 1. On the 4th and 5th year : Biological markers 1 time per year | 32 | 9 |  |  | 1 | 1 | 1 |  | 2 | 7 | 20 | N |
|  |  | 1. On the 4th year, ultrasound abdominopelvic instead of abdominopelvic scanner | 32 | 8 | 1 | 1 | 1 | 1 | 5 |  | 4 | 9 | 10 | N |
|  |  | 1. Stop surveillance after 5 years of follow-up | 32 | 8 |  |  | 1 | 1 | 2 |  | 5 | 10 | 13 | N |
| Non-seminoma | surveillance alone | 1. On the 1st year : Physical examination and biological markers 4 times per year (relevance) | 32 | 9 | 1 |  |  | 1 | 2 |  | 1 | 4 | 23 | N |
|  |  | 1. On the 1st year : Physical examination and biological markers 4 times per year (feasibility) | 32 | 9 | 1 | 1 |  |  |  | 2 | 1 | 5 | 22 | N |
|  |  | 1. On the 2nd year : Physical examination and biological markers 4 times per year (relevance) | 32 | 9 |  | 1 |  |  | 3 | 2 | 1 | 6 | 19 | N |
|  |  | 1. On the 2nd year : Physical examination and biological markers 4 times per year (feasibility) | 32 | 9 | 1 | 1 |  | 2 |  | 1 |  | 7 | 20 | N |
|  |  | 1. On the 4th and 5th year : Physical examination and biological markers 1 time per year | 32 | 8 |  |  | 1 | 1 | 3 |  | 4 | 10 | 13 | N |
|  |  | 1. Chest x-ray instead of thoracic scanner | 32 | 5 | 5 | 2 | 5 | 1 | 6 | 2 | 3 | 3 | 5 | N |
|  |  | 1. On the 1st and 2nd year : chest x-ray 2 times per year | 32 | 8.5 | 1 |  |  |  | 1 |  | 5 | 9 | 16 | N |
|  |  | 1. No systematic chest imaging after the 3rd year | 32 | 7 | 1 | 1 | 2 | 2 | 3 |  | 9 | 8 | 6 | N |
|  |  | 1. On the 2nd year : Abdominopelvic imaging 1 time per year | 32 | 6 | 2 | 2 | 4 | 2 | 5 | 2 | 2 | 6 | 7 | N |
|  |  | 1. On the 4th year : no systematic imaging | 32 | 8 |  | 1 | 1 | 1 | 4 |  | 7 | 10 | 8 | N |
|  |  | 1. Stop surveillance after 5 years of follow-up | 32 | 9 |  |  | 1 | 1 | 2 |  | 4 | 6 | 18 | N |
|  | Non-seminoma on surveillance after chemotherapy | 1. On the 1st year : Physical examination and biological markers 4 times per year (relevance) | 32 | 9 | 2 |  | 1 |  | 1 |  | 4 | 7 | 17 | N |
|  |  | 1. On the 1st year : Physical examination and biological markers 4 times per year (feasibility) | 32 | 9 | 1 |  | 1 | 1 |  | 1 | 1 | 8 | 19 | N |
|  |  | 1. On the 2nd year : Physical examination and biological markers 4 times per year (relevance) | 32 | 8 | 1 | 1 | 2 |  | 1 | 1 | 2 | 9 | 15 | N |
|  |  | 1. On the 2nd year : Physical examination and biological markers 4 times per year (feasibility) | 32 | 9 | 1 | 1 | 2 |  |  | 1 |  | 8 | 19 | N |
|  |  | 1. On the 4th and 5th year, if LVI negative, physical examination and biological markers 1 time per year | 32 | 8 |  |  |  |  |  | 1 | 6 | 12 | 13 | R |
|  |  | 1. On the 4th and 5th year, if LVI positive, physical examination and biological markers 2 times per year | 32 | 9 | 1 |  | 1 |  | 2 |  | 3 | 7 | 18 | N |
|  |  | 1. Chest x-ray instead of thoracic scanner | 32 | 5 | 3 | 2 | 5 | 1 | 8 | 1 | 1 | 6 | 5 | N |
|  |  | 1. On the 1st and 2nd year, if LVI negative, chest imaging 1 time per year | 32 | 8 | 2 |  |  |  | 3 |  | 7 | 13 | 7 | N |
|  |  | 1. On the 1st and 2nd year, if LVI positive, chest imaging 2 times per year | 32 | 8 | 1 |  |  |  | 2 |  | 3 | 12 | 14 | N |
|  |  | 1. After the 3rd year, chest imaging 1 time per year | 32 | 8 | 1 |  | 1 |  |  |  | 2 | 13 | 15 | N |
|  |  | 1. On the 1st and 2nd year, if LVI negative, abdominopelvic imaging 1 time per year | 32 | 8 |  |  | 1 | 1 | 2 |  | 3 | 14 | 11 | N |
|  |  | 1. On the 1st and 2nd year, if LVI positive, abdominopelvic imaging 2 times per year | 32 | 9 | 1 |  |  |  | 1 |  | 3 | 8 | 19 | N |
|  |  | 1. On the 4th year : no systematic imaging | 32 | 8 |  | 1 | 1 | 2 | 2 | 1 | 5 | 12 | 8 | N |
|  |  | 1. Stop surveillance after 5 years of follow-up | 32 | 8.5 |  |  | 1 | 1 | 4 |  | 2 | 8 | 16 | N |

Experts are asked to rate each item according to its relevance for prescription (Abbreviations: C, consensus; S, strong; R, Relative; N, absence of consensus
